# Dynamical Behaviors of Rb-E2F Pathway Including Negative Feedback Loops Involving miR449

**DOI:** 10.1371/journal.pone.0043908

**Published:** 2012-09-18

**Authors:** Fang Yan, Haihong Liu, Junjun Hao, Zengrong Liu

**Affiliations:** 1 Department of Mathematics, Shanghai University, Shanghai, P. R. China; 2 Institute of System Biology, Shanghai University, Shanghai, P. R. China; 3 Department of Mathematics, Yunnan Normal University, Kunming, P. R. China; Institut Jacques Monod, France

## Abstract

MiRNAs, which are a family of small non-coding RNAs, regulate a broad array of physiological and developmental processes. However, their regulatory roles have remained largely mysterious. E2F is a positive regulator of cell cycle progression and also a potent inducer of apoptosis. Positive feedback loops in the regulation of Rb-E2F pathway are predicted and shown experimentally. Recently, it has been discovered that E2F induce a cluster of miRNAs called miR449. In turn, E2F is inhibited by miR449 through regulating different transcripts, thus forming negative feedback loops in the interaction network. Here, based on the integration of experimental evidence and quantitative data, we studied Rb-E2F pathway coupling the positive feedback loops and negative feedback loops mediated by miR449. Therefore, a mathematical model is constructed based in part on the model proposed in Yao-Lee et al. (2008) and nonlinear dynamical behaviors including the stability and bifurcations of the model are discussed. A comparison is given to reveal the implication of the fundamental differences of Rb-E2F pathway between regulation and deregulation of miR449. Coherent with the experiments it predicts that miR449 plays a critical role in regulating the cell cycle progression and provides a twofold safety mechanism to avoid excessive E2F-induced proliferation by cell cycle arrest and apoptosis. Moreover, numerical simulation and bifurcation analysis shows that the mechanisms of the negative regulation of miR449 to three different transcripts are quite distinctive which needs to be verified experimentally. This study may help us to analyze the whole cell cycle process mediated by other miRNAs more easily. A better knowledge of the dynamical behaviors of miRNAs mediated networks is also of interest for bio-engineering and artificial control.

## Introduction

MicroRNAs (miRNAs) have been demonstrated to play crucial roles both in prokaryotes and eukaryotes [1], [2]. Their importance is suggested in [1]–[3] by (i) the predictions that each miRNA targets hundreds of genes and that the majority of protein-coding genes are miRNA targets [4]–[8], (ii) their abundance, with some miRNAs expressed as highly as 50,000 copies per cell [9], and (iii) their sequence conservation, with some miRNAs conserved from sea urchins to humans [10]. MiRNAs can regulate a large variety of cellular processes, from differentiation and proliferation to apoptosis [11]–[15], by determining how and when genes turn on and off. Thus, from a biological point of view, miRNAs are challenging objects to study as they regulate cohorts of target genes, which are not readily identified. From a therapeutical point of view, miRNAs are highly interesting as several studies have demonstrated the power of miRNAs as biomarkers and initial preclinical studies have established that miRNAs may be therapeutically targeted in vivo [16].

MiRNAs regulate protein synthesis in the cell cytoplasm by promoting target mRNAs' degradation and/or inhibiting their translation. It is thought that the primary role of miRNAs is to modulate or fine-tune the dynamics of regulatory networks [17]–[20]. The significance of this role is now increasingly recognized as there are now many reported cases in which miRNA deregulation in a broad spectrum of diseases including all major cancers [21]. The regulatory roles of miRNAs have been a subject of research for the last several years, both experimentally and theoretically [6]–[10]. Although some of the miRNAs have been well studied, the information about possible functions and biological significance of miRNAs still need to be fully understood due to the diversity of mechanisms by which miRNAs may regulate biological processes. Here, we focus on miR449, which has been identified significantly down-regulated or lost in gastric cells, testicular cancer cells as well as in lung adenocarcinoma cell line[21]–[24]. Its reintroduction into these cancer cell lines leads to inhibition of cell proliferation and induces senescence and apoptosis by targeting different cell cycle regulators. Hence, these studies further underly that miR449 may act as a tumor suppressor [21], [22].

The E2F family is best known for its critical role in cell proliferation. It is intimately connected to cell proliferation by coordinating a large group of genes involving G1 to S transition. In normal cells the E2F activity is tightly controlled at multiple levels. Deregulated E2F activity occurs in the vast majority of human tumors through several different mechanisms. These changes often lead to the inappropriate activation of E2F and its transcriptional target genes that further result in improper cell cycle control and unrestricted proliferation. Thus, repressing E2F activity is the key mechanism to exert the tumor-suppresive function [25]. Although the detailed mechanism of posttranscriptional regulation by miRNAs is not fully understood, evidence for the functional roles of miRNAs is accumulating. Recent study demonstrates that miRNAs mainly control transcripts coding for proteins involved in cell damage responses, cell cycle control, inflammation and cancer pathways [22]. Interestingly, it has been discovered that E2F strongly upregulate miR449 [21], [22], [26]. In turn, E2F is inhibited by miR449 through regulating different transcripts, thus forming negative feedback loops in the interaction network. This leads to following natural questions: what will occur by adding these negative feedback loops into Rb-E2F pathway and how does miR449 fine-tune the dynamics in this cancer network.

To address these questions in this paper, a mathematical model is constructed based in part on the model proposed in Yao-Lee et al. (2008) and quantitative comparison of dynamical features associated with or without miR449 regulation is given. They largely differ in the onset of bistability and oscillations and further affects E2F activity and G1/S transition. It is expected that the difference will generate a detailed and precise insight of mi449 mediated regulation. In agreement with experimental observations, the model verified that miR449 plays a critical role in regulating the cell cycle progression and provides a twofold safety mechanism to avoid excessive E2F-induced proliferation by cell cycle arrest and apoptosis. Moreover, bifurcation analysis of the model predicts that different mechanisms are employed by miR449 to negative regulate three different transcripts, which needs to be verified experimentally. These results are important for understanding the dynamics and functions of miRNAs and provide clues for therapeutic manipulation of Rb-E2F pathway in the treatment of cancer.

## Results

In this research, particular attentions are payed to the bistability and oscillations. Bistability, the capacity to achieve two alternative internal states in response to different stimuli, exists ubiquitously in synthetic and natural biomolecular networks and has fundamental biological significance [27]–[30]. Cells can switch between two internal states to accommodate environmental and intercellular conditions owing to regulatory interactions among cellular components. Oscillatory behaviors are generated by complex interactions among genes, proteins and metabolites. They are used to control every aspect of cell physiology, from signalling, motility and development to growth, division and death [30]–[32]. Hopf bifurcations, including supercritical and subcritical Hopf bifucations, are the key principle of designing biochemical oscillators. In the following, numerical simulation and bifurcation analysis in our paper were carried out with XPPAUT. Moreover, XPPAUT codes are available in the Supporting Information File S1, S2, S3, S4, S5, S6, S7, S8, S9, S10, S11, S12, S13, 14.

### Modeling Rb-E2F pathway mediated by miR449

**Figure 1:**
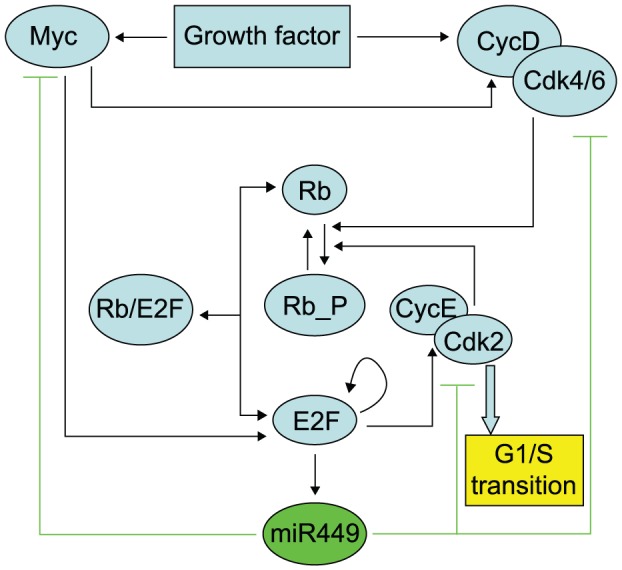
Rb-E2F pathway mediated by miR449. Rb-E2F circuit coupling the positive feedback loops and negative feedback loops mediated by miR449. E2F represents all E2F activators (E2F1, E2F2 and E2F3a). Rb represents all pocket proteins (Rb, p130 and p107). MiR449 represents miR449 family (miR449a, miR449b and miR449c).

We focus on Rb-E2F pathway involved multiple positive feedback loops and negative feedback loops mediated by miR449. The network that we considered in this paper is shown schematically in Figure 1. E2F is a transcriptional factor that can activate genes encoding proteins involved in DNA replication and cell cycle progression [25], [33]–[35], and is essential in the regulation of cell proliferation [25], [36], [37]. In quiescent cells, E2F is bound to and repressed by retinoblastoma protein (Rb), a tumor suppressor protein that is dysfunctional in several major cancers. With sufficient growth stimulation, phosphorylation by Myc-induced cyclin D (CycD)-Cdk4,6 removes Rb repression; Myc also induces E2F transcription. Subsequently, E2F activates the transcription of CycE, which forms a complex with Cdk2 to further remove Rb repression by phosphorylation, establishing a positive-feedback loop. E2F also activates its own transcription, constituting another positive-feedback loop [25]. An intriguing addition to the already complex regulatory mechanism of Rb-E2F network is the recent discovery that miR449 modulate E2F activity [21], [22], [26], [38]. It has been demonstrated that E2F strongly upregulate the expression of miR449. In turn, E2F is inhibited by miR449 through regulating different transcripts, thus forming negative feedback loops. One of the miR449 regulation is that it directly affects level of its target transcript Myc and therefore lower E2F concentration. The other one is directly affects E2F inducer Cdk6 and CycE. Besides these two scenarios, miR449 also target Cdc25A, a oncoprotein that positively regulate E2F and is required for progression from G1 to the S phase of the cell cycle. It is competent to activate the G1/S cyclin-dependent kinases Cdk4 and Cdk2 by removing inhibitory phosphate groups from adjacent tyrosine and threonine residues. To some extent because the negative effect of miR449 on Cdc25A can be considered by choosing different inhibitory parameters of miR449 on CycD-Cdk4/6 and CycE-Cdk2 complex, our network does not explicitly include the negative effect of miR449 on Cdc25A.

Mathematical models of cell cycle regulation including Rb-E2F pathway have been previously proposed [25], [39]–[42], however, the effects of miRNAs on this network have not been investigated. Meanwhile, miRNAs are presented as a family of critical regulators of almost all cellular processes. So investigation on the regulatory mechanisms of miRNAs is urgent and meaningful especially for therapeutic manipulation in the treatment of cancer. In the present paper, we dedicate to explore the regulatory effects of miR449 on Rb-E2F pathway. According to the scheme of Figure 1, the dynamical relations of the network are characterized by following nonlinear ordinary differential equations (ODEs):
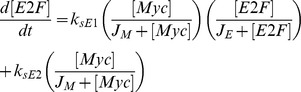


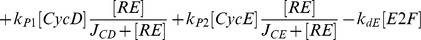


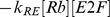


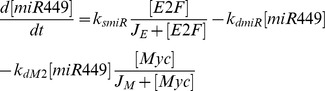


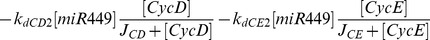


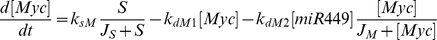


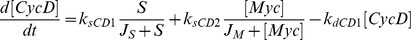


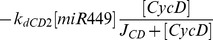


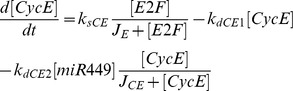


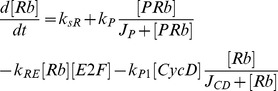


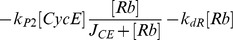


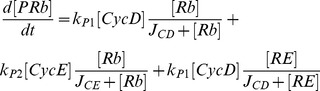


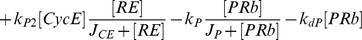


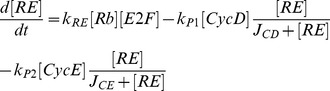


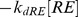
where [E2F], [miR449], [Myc], [CycD], [CycE], [Rb], [PRb] and [RE] represent their concentrations of E2F, miR449, Myc, CycD-Cdk4/6 complex, CycE-Cdk2 complex, Rb, phosphorylated Rb and Rb/E2F complex, respectively. And 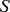 is intensity of growth factor. Our model is based on that of Yao-Lee et al. (2008), with one key difference. That is the effect of miR449 on Rb-E2F pathway not involved in the model proposed by Yao-Lee et al. has been taken as key consideration in the present paper. This mathematical model can be directly translated from the Figure 1. Dimerization and transformation are modeled as elementary reactions. Moreover, transcription/activation and inhibition are modeled using the Hill functions. Here, model parameters were chosen based on the literature whenever possible [25], and on the biochemical constraints [21], [22], [26], [42] to give simulations and bifurcation diagrams that are consistent with known dynamical behaviors of miR449 and Rb-E2F pathway. In the following simulations, all the values of employed parameters are shown in Table 1 unless specified elsewhere.

**Table 1:** Parameters values for the mathematical model.

Rate constant	Description	Value	Reference
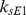	Rate constant of E2F production by a synergy between Myc and E2F	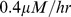	[25]
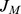	Michaelis constant of Myc in transactivation	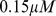	[25]
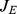	Michaelis constant of E2F in transactivation	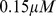	[25]
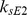	Rate constant of basal E2F production by Myc	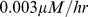	[25]
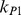	Phosphorylation rate of Rb by CycD-Cdk4/6 complex	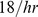	[25]
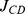	Michaelis constant of Rb in CycD-Cdk4/6-dependent phosphorylation	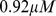	[25]
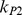	Phosphorylation rate of Rb by CycE-Cdk2 complex	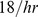	[25]
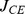	Michaelis constant of Rb in CycE-Cdk2-dependent phosphorylation	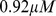	[25]
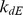	Degradation rate of E2F	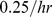	[25]
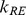	Associate rate of Rb and E2F	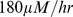	[25]
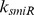	Rate constant of miR449 production by E2F	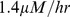	[48]
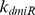	Degradation rate of miR449	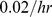	[48]
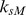	Rate constant of Myc production by growth factor	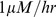	[25]
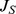	Michaelis constant of growth factor	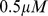	[25]
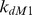	Degradation rate of Myc	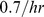	[25]
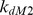	Inhibition rate of Myc by miR449	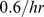	Estimated
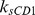	Rate constant of CycD-Cdk4/6 complex production by growth factor	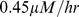	[25]
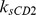	Rate constant of CycD-Cdk4/6 complex production by Myc	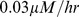	[25]
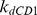	Degradation rate of CycD-Cdk4/6 complex	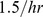	[25]
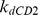	Inhibition rate of CycD-Cdk4/6 complex by miR449	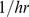	Estimated
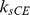	Production rate of CycE-Cdk2 complex by E2F	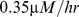	[25]
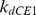	Degradation rate of CycE-Cdk2 complex	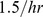	[25]
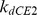	Inhibition rate of CycE-Cdk2 complex by miR449	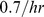	Estimated
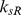	Basal production rate of Rb	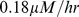	[25]
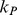	Dephosphorylation rate of Rb	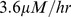	[25]
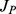	Michaelis constant of PRb dephosphorylation	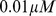	[25]
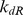	Degradation rate of Rb	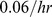	[25]
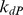	Degradation rate of PRb	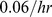	[25]
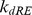	Degradation rate of Rb/E2F complex	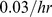	[25]

### The effects of miR449 on Rb-E2F pathway

When analyzing qualitative information about Rb-E2F pathway, we choose the growth factor 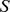 as a governing parameter because it is the key parameter responsible for the G1/S transition in mammalian and plant cell cycle. Moreover, E2F plays a major role during the G1/S transition and CycE-Cdk2 as an output of Rb-E2F pathway is an important mark of cell cycle transition from 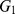 to 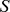 phase. Thus, in order to investigate the dynamical potential of miR449 in regulation of cell cycle, we will next examine and compare the dynamical behaviors of E2F and CycE-Cdk2 complex without regulation of miR449 or under this regulation.

In the following, if the concentration of E2F lingers on a high stable level state, then the cell cycle is at a state of uncontrolled cell proliferation [43]–[46]. When the concentration of E2F is at a state of sustained oscillations with high amplitudes, then the cell cycle is at a state of normal cell cycle process [46]. If the concentration of E2F is at a state of sustained oscillations with low amplitudes or a low stable level state, then the cell cycle is arrested in G1 phase [46]. While the concentration of E2F is at a state of relatively high level (lower than the high stable state and higher than the low stable state), then the cell undergoes apoptosis [46], [47]. Note that, in the following bifurcation diagrams, the red and black lines respectively represent stable and unstable steady states, green dots are the maxima and minima of the stable limit cycles, while blue open circles denote the maxima and minima of the unstable limit cycles, and 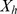 is the horizontal coordinate of point 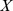.

**Figure 2:**
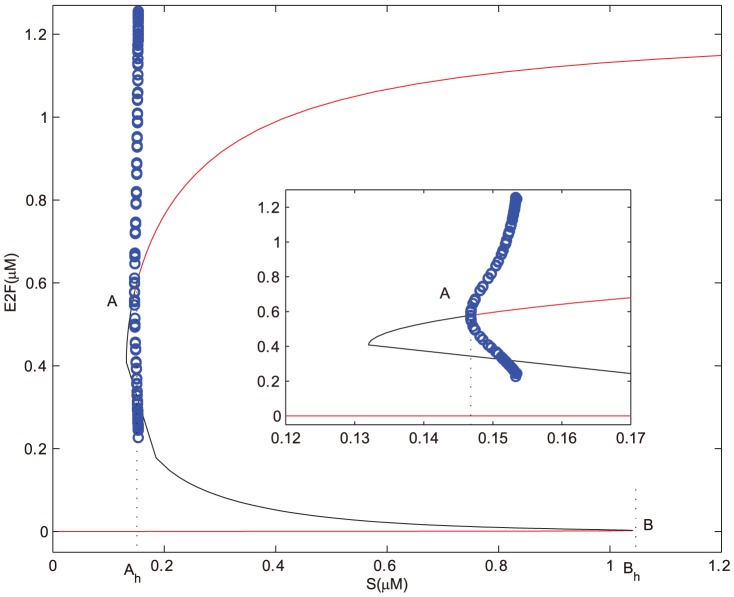
Bifurcation diagram of [E2F] with 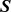 as a control parameter at 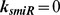. Set the AUTO axes to run from 0 to 1.2 along the x-axis and from −0.05 to 1.25 along the y-axis. The initial values for the simulation are 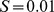, [E2F] = 0, [MiR449] = 0.004, [Myc] = 0.0280, [Cdk6] = 0.0090, [CycE] = 0, [Rb] = 2.9918, [PRb] = 0.0004, [RE] = 0.0157.

**Figure 3:**
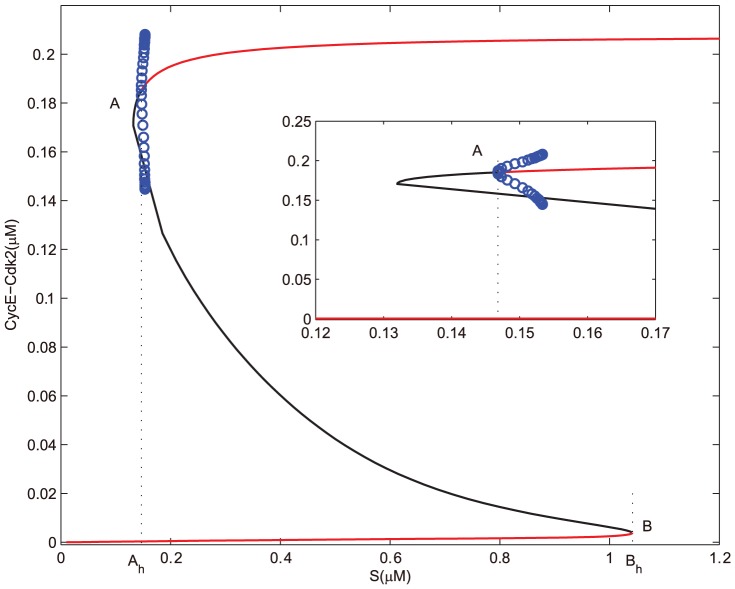
Bifurcation diagram of [CycE-Cdk2] with S as a control parameter at 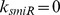. Set the AUTO axes to run from 0 to 1.2 along the x-axis and from −0.001 to 0.22 along the y-axis. Initial values as in Fig. 2.

Without the effects of miR449, bifurcation diagrams of E2F and CycE-Cdk2 complex concentrations versus the growth factor 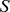 are obtained in Figure 2 and 3, where 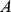 is a subcritical Hopf bifurcation point and 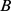 is a saddle-node bifurcation point. Contrasting Figure 2 with Figure 3, it can be seen that E2F and CycE-Cdk2 are so sensitive to growth factor 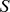 that 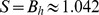 is sufficient to activate them. And the dynamical behaviors of both E2F and CycE-Cdk2 are almost synchronous, that is because CycE is produced by E2F. From Figure 2 and 3, there exists an unique stable steady state corresponding to the quiescent state when 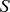 is small enough. As 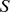 increases gradually, bistability occurs in the range starting from 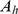 to 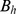. Particularly, a subcritical Hopf bifurcation appears at 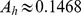, which leads to a series of sustained oscillations of both E2F and CycE-Cdk2 corresponding to the normal cell cycle. When the growth factor 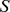 keeps on going out of the bistable range, these oscillations vanish and then E2F and CycE-Cdk2 step into their high stable steady states. Meanwhile, the value of the high stable state in Figure 2 is almost close to the maximum of the oscillation. It is more obvious especially for CycE-Cdk2 as shown in Figure 3. This means that if 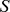 surpasses the bistable range, cell undergoes excessive proliferation which will leads to uncontrolled cell proliferation.

**Figure 4:**
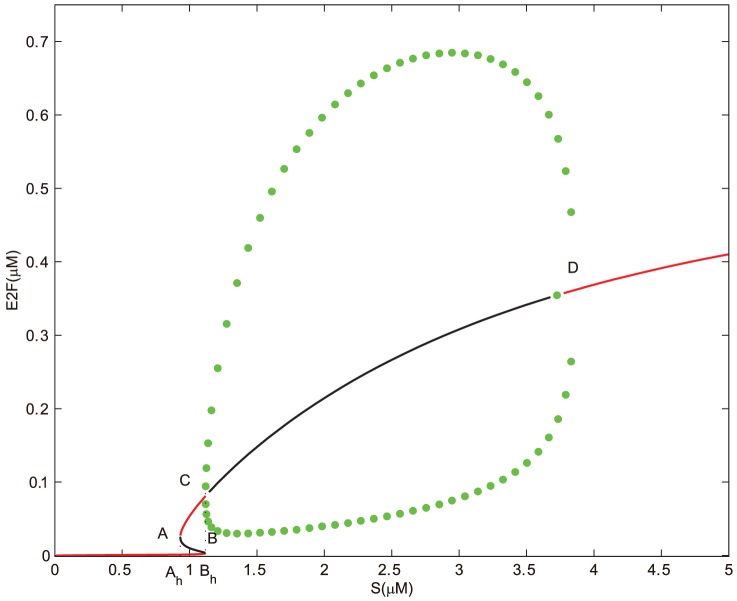
Bifurcation diagram of [E2F] with S as a control parameter at 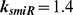. Set the AUTO axes to run from 0 to 5 along the x-axis and from 0 to 0.75 along the y-axis. Initial values as in Fig. 2.

**Figure 5:**
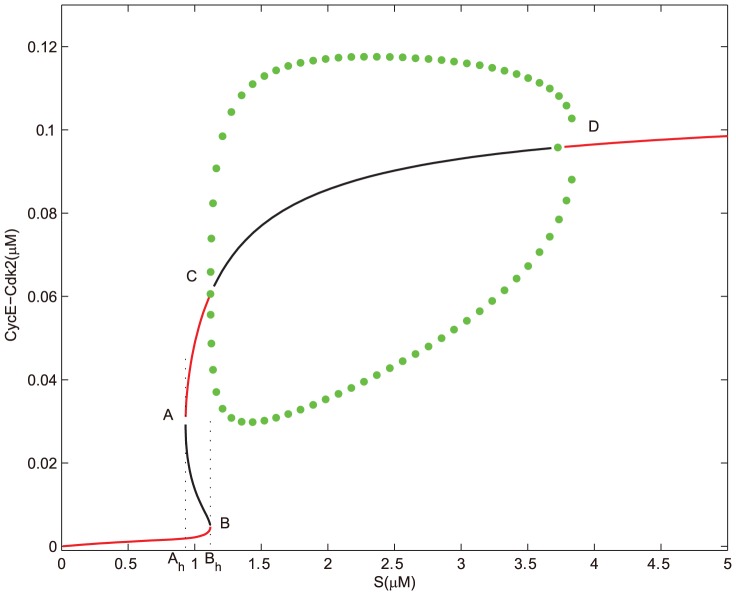
Bifurcation diagram of [CycE-Cdk2] with 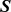 as a control parameter at 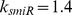. Set the AUTO axes to run from 0 to 5 along the x-axis and from −0.001 to 0.13 along the y-axis. Initial values as in Fig. 2.

Under the regulation of miR449, bifurcation diagrams for the concentrations of E2F and CycE-Cdk2 complex corresponding to the growth factor 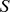 are shown in Figure 4 and 5. To highlight distinct features associated with the one deregulation of miR449, we consider similar parameters to above except 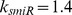 instead of 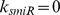. Obviously, both 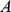 and 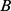 are saddle-node bifurcation points; 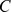 and 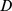 are supercritical Hopf bifurcation points. From Figure 4 and 5, it can be seen that there exists an unique stable steady state corresponding to the quiescent state if 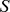 is small enough. As 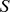 increases gradually, bistability occurs in the range starting from 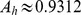 to 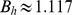. A lower and a higher stable steady state coexist. However, when 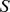 keeps on going out of the range, the higher stable steady state loses its stability and a supercritical Hopf bifurcation appears at 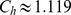, which leads to a stable branch of limit cycle corresponding to a series of sustained oscillations. When 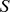 is increased to 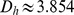, these oscillations vanished. In fact, the amplitudes of these oscillations near 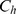 and 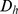 are low, which indicates the G1/S transition does not happen and corresponds the cell cycle arrest. While at an intermediate 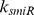 value between 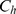 and 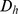, one can notice that the amplitudes of these oscillations are high, which may corresponds to the normal cell cycle process. When 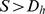, E2F steps into the highest stable state which monotonically increases at the beginning and then tends almost to the horizontal line. While the concentration of CycE-Cdk2 reaches its saturation state more quickly. But it is much lower than the maximum of the oscillations corresponding to the normal cell cycle. This means that the cell undergoes apoptosis instead of proliferation and cell cycle arrest.

**Figure 6:**
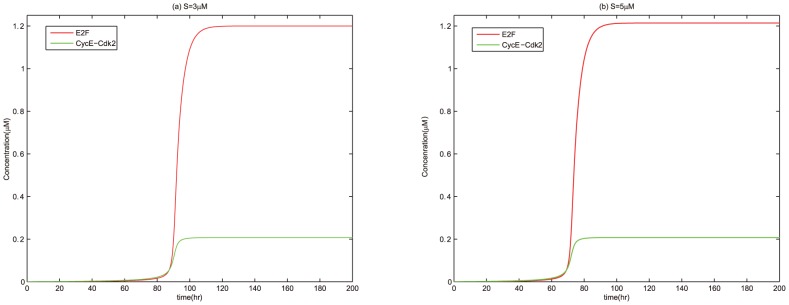
Time courses of [E2F] and [CycE-Cdk2] at 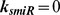. Assume initial conditions are [E2F] = 0, [MiR449] = 0, [Myc] = 0, [Cdk6] = 0, [CycE] = 0, [Rb] = 0.55, [PRb] = 0, [RE] = 0. Set the Viewaxes run from 0 to 200 along the x-axis and from 0 to 1.25 along the y-axis.(a) 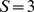; (b) 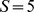.

**Figure 7:**
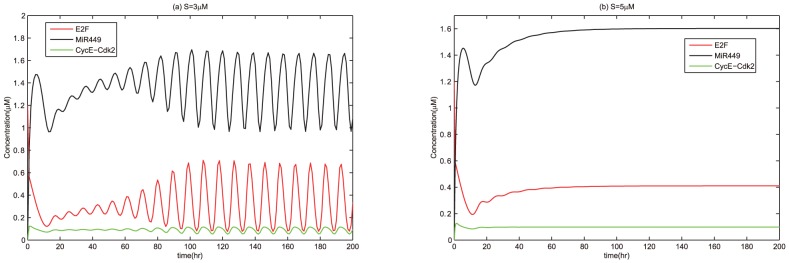
Time courses of [E2F], [CycE-Cdk2] and [MiR449] at 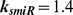. Assume initial conditions are [E2F] = 1.2, [MiR449] = 0, [Myc] = 0, [Cdk6] = 0, [CycE] = 0, [Rb] = 0.55, [PRb] = 0, [RE] = 0. (a) 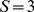. Set the Viewaxes run from 0 to 200 along the x-axis and from 0 to 2 along the y-axis; (b) 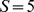. Set the Viewaxes run from 0 to 200 along the x-axis and from 0 to 1.7 along the y-axis.

For thorough testify the dynamical potential of miR449 in regulation of cell cycle, we also compared the time courses of E2F and CycE-Cdk2 complex without regulation of miR449 or under this regulation as show in Figure 6 and 7. Without the regulation of miR449, dynamical processes of E2F and CycE-Cdk2 complex with 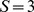 and 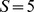 are show in Figure 6(a) and (b), respectively. It can be seen that the concentration of E2F and CycE-Cdk2 complex keep in a low level at the beginning and suddenly jump to a high level. The only difference is that the jump is occurs at 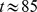 in Figure 6(a) and 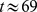 in Figure 6(b). That is, when 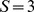, the cell transits from a quiescent state to an excessive E2F-induced proliferation state at 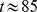. And when 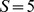, this transition happens at 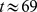. Under the regulation of miR449, the time courses of E2F and CycE-Cdk2 complex with 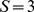 and 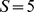 are show in Figure 7(a) and (b), respectively. The initial state of E2F is an inappropriate activated state with [E2F] = 1.2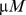. It was easy to see that there are essential differences between the two diagrams. The concentration of E2F is driven from a high level state to a sustained oscillatory state as shown in Figure 7(a). Specially, the concentration of E2F quickly drops at the beginning due to strong up-regulation of E2F on miR449 and heavy down-regulation of miR449 on E2F. And then the concentrations of E2F, CycE-Cdk2 and miR449 undergo sustained oscillations with low amplitudes in the primary stage and high amplitudes later due to the effects of the negative feedback loops between E2F and miR449. This means that the cell cycle is quickly driven from an excessive E2F-induced proliferation state to a cell cycle arrest state, which rendered the cell to repair DNA damage. After all the damage is repaired, the concentrations of E2F, CycE-Cdk2 and miR449 undergo sustained oscillations with high amplitudes corresponding to a normal proliferation state. In Figure 7(b), the concentration of E2F are driven from a high level state to a relatively high level state compared with the low level in Figure 6. This means that miR449 has the ability to drive cell cycle from excessive E2F-induced proliferation state to a state of apoptosis. Therefore, miR449 plays a dual role in regulation of cell cycle. Specially, miR449 drives the cell cycle from an excessive E2F-induced proliferation state returning to a normal proliferation state in a moderate stimulation of 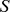 by promoting cell cycle arrest, but leads to apoptosis for a high stimulation of 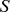.

### The effects of miR449 on E2F activity

**Figure 8:**
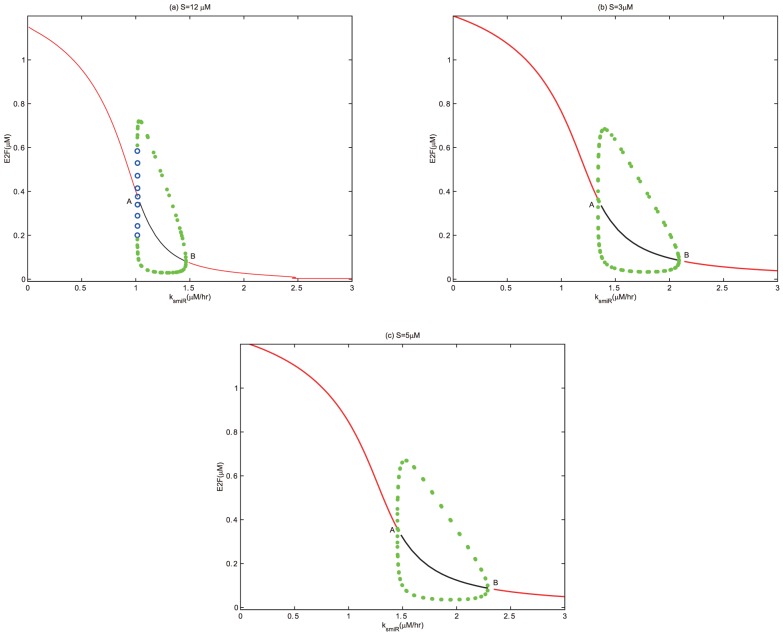
Bifurcation diagrams of [E2F] with 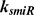 as a control parameter. Set the AUTO axes to run from 0 to 3 along the x-axis and from −0.01 to 1.2 along the y-axis. (a) 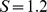 with initial values as 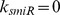, [E2F] = 1.1489, [MiR449] = 0, [Myc] = 1.0084, [Cdk6] = 0.2292, [CycE] = 0.2064, [Rb] = 0.0175, [PRb] = 2.5921, [RE] = 0.7793; (b) 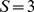 with initial conditions as 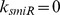, [E2F] = 1.1998, [MiR449] = 0, [Myc] = 1.2245, [Cdk6] = 0.2750, [CycE] = 0.2074, [Rb] = 0.0167, [PRb] = 2.6588, [RE] = 0.6489; (c) 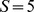 with initial values as 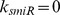, [E2F] = 1.2137, [MiR449] = 0, [Myc] = 1.2987, [Cdk6] = 0.2907, [CycE] = 0.2077, [Rb] = 0.0165, [PRb] = 2.6767, [RE] = 0.6136.

In order to further explain the effectiveness of miR449 in inhibiting E2F activity, we consider the effects of variations in the parameter 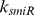 (a rate constant of miR449 production) and 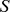 on the concentration of E2F. The results in Figure 8 show that the concentration of E2F is sensitive to the variation of 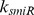 and not particularly sensitive to the variation of 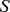. Therefore, E2F activity can be significantly affected by the regulation of miR449.

As shown in Figure 8, the concentration of E2F decreases with 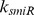 because a larger rate of miR449 production means lower E2F activity. The stability of the equilibrium can be changed with the variation in 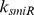. In absence of miR449 regulation, i.e., 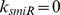, the unique equilibrium is stable. E2F settles on a high stable steady state corresponding to an excessive E2F-induced proliferation state. When miR449 is introduced, i.e., 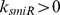, E2F drops rapidly with increasing 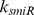. It means that the cell cycle is quickly driven from an excessive E2F-induced proliferation state to a cell cycle arrest state (because the concentration of E2F is lower than the maximum of the oscillations corresponding to the normal cell cycle in the left-neighborhood of point A). As 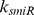 increases gradually, the stability of the equilibrium transforms from stable to unstable, meanwhile sustained oscillations appear. In fact, one can notice that the amplitudes of these oscillations near 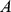 and 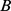 are low, which indicates that the G1/S transition does not happen and corresponds to the cell cycle arrest. However, the amplitudes of these oscillations near a certain value of 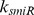 are high (specially, 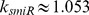 in Figure 8(a), 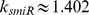 in Figure 8(b), 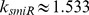 in Figure 8(c)), which may corresponds to the normal cell cycle process. When the parameter 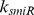 keeps on going out of the oscillatory range, oscillations vanish and the unique equilibrium regains its stability. E2F steps into a low stable steady state corresponding to the state of cell cycle arrest. If the value of 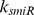 is large enough, the activity of E2F is almost completely inhibited and cell growth is permanently arrested in the G1 phase.

However, it is worth noting that this inhibitory process of miR449 on E2F has a little difference for different level of 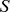 in the middle range of 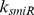. For example, in Figure 8(a) with 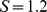, there exist a subcritical Hopf bifurcation 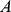 at 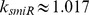 and a supercritical Hopf bifurcation 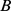 at 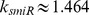. Stable oscillation and equilibrium may coexist for values of 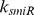 in the interval enclosed by 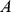 and a fold limit cycle bifurcation (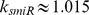) due to the occurrence of the subcritical Hopf bifurcation. In Figure 8(b) with 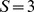, there exist two supercritical Hopf bifurcations 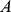 at 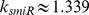 and 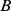 at 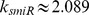 as 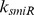 increases. The first one is supercritical, resulting in a stable branch of limit cycles. While the second one is also supercritical because the equilibrium branch loses the stability going left and the periodic orbit branch goes left too. Similar to Figure 8(b), there exist two supercritical Hopf bifurcations 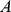 at 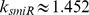 and 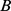 at 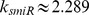 in Figure 8(c) with 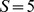. Comparing the three diagrams, one can notice that the larger 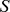 is, the larger 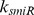 and the longer oscillation range will be needed to completely inhibit E2F activity. In other words, the more growth factor, the more miR449 is needed to inhibit the activity of E2F.

**Figure 9:**
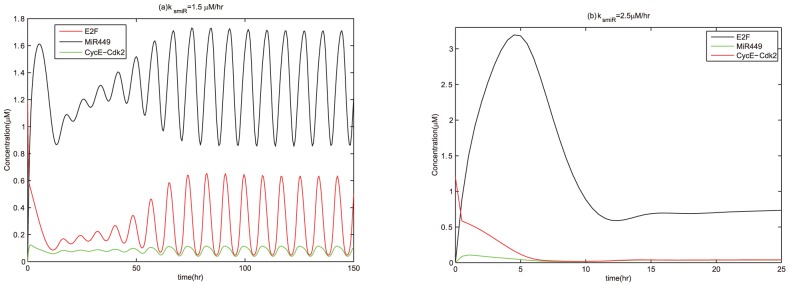
Time courses of [E2F], [CycE-Cdk2] and [MiR449] at 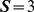. Assume initial conditions are [E2F] = 1.2, [MiR449] = 0, [Myc] = 0, [Cdk6] = 0, [CycE] = 0, [Rb] = 0.55, [PRb] = 0, [RE] = 0. (a) 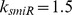. Set the Viewaxes run from 0 to 150 along the x-axis and from 0 to 1.8 along the y-axis; (b) 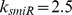. Set the Viewaxes run from 0 to 25 along the x-axis and from 0 to 3.3 along the y-axis.

Therefore, no matter how large 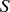 is, a value of 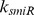, which always exists, is sufficient to inhibit the concentration of E2F from a high stable steady state to a state of sustained oscillations whose amplitudes undergo a change from low to high and then again to low. In other words, no matter how large 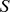 is, there always exists a value of 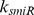 that is sufficient to drive the cell cycle from an excessive E2F-induced proliferation state (high stable steady state with high concentration) to a normal proliferation state (sustained oscillations with high amplitudes) by promoting the cell cycle arrest (oscillations with low amplitudes or high stable steady state with low concentration in the left-neighborhood of point A). For example, assuming that 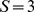 and the initial state of E2F is an inappropriate activated state with [E2F] = 1.2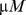, there exists 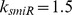 that is sufficient to drive E2F from a high stable steady state to a sustained oscillatory state as shown in Figure 9(a). It can be seen that the concentration of E2F quickly drops at the beginning due to strong positive regulation of E2F on miR449 and strong inhibitory regulation of miR449 on E2F. And then the concentrations of E2F, CycE-Cdk2 and miR449 undergo sustained oscillations with low amplitudes in the primary stage and high amplitudes later due to the effects of the negative feedback loops between E2F and miR449. This means that the cell cycle is quickly driven from an excessive E2F-induced proliferation state to a cell cycle arrest state, which rendered the cell to repairing DNA damage. After all the damage is repaired, the concentrations of E2F, CycE-Cdk2 and miR449 undergo sustained oscillations with high amplitudes corresponding to a normal proliferation state. Moreover, no matter how large 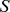 is, a value of 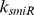, which always exists, is sufficient to inhibit the concentration of E2F from a high stable steady state to a low stable steady state even to zero. In other words, no matter how large 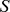 is, there always exists a value of 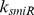 that is sufficient to drive the cell cycle from excessive E2F-induced proliferation state to a state of cell cycle permanent arrested in the G1 phase (senescence). For example, there exists 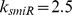 that is sufficient to drive cell cycle from excessive E2F-induced proliferation state to a state of cell cycle permanent arrested in the G1 phase as shown in Figure 9(b). Therefore, E2F is so sensitive to the rate constant of miR449 production and miR449 remarkably inhibits E2F activity. These numerical results and analysis are coherent with the experiments. Thus, miR449 acts as an effective tumor suppressor.

### Different repressions of miR449 on E2F via three different targets

**Figure 10:**
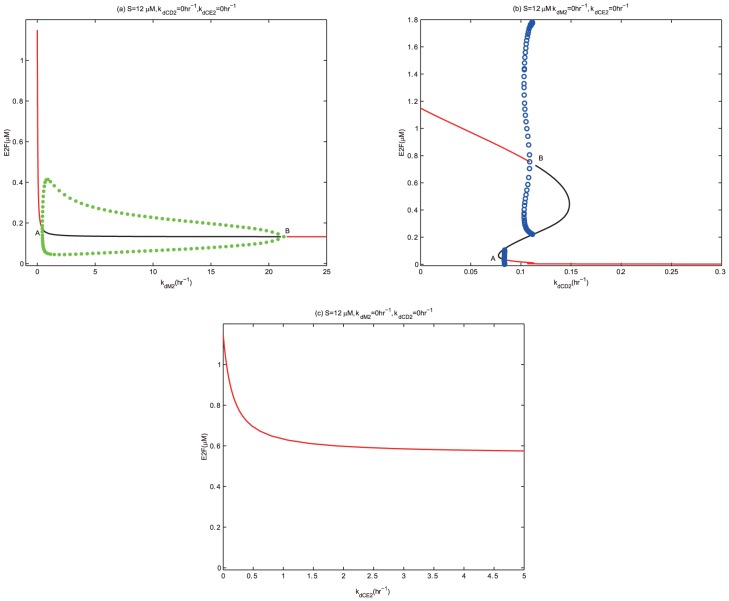
Bifurcation diagrams of [E2F] with inhibition rates of miR449 on three targets as control parameters. Set up initial conditions as 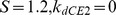, [E2F] = 1.1489, [MiR449] = 61.9160, [Myc] = 1.0084, [Cdk6] = 0.2292, [CycE] = 0.2064, [Rb] = 0.0175, [PRb] = 2.5929, [RE] = 0.7793. (a) Bifurcation diagram of [E2F] with 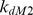 as a control parameter at 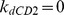 and 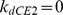. Set the AUTO axes to run from −1 to 25 along the x-axis and from −0.01 to 1.2 along the y-axis; (b) Bifurcation diagram of [E2F] with 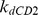 as a control parameter at 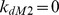 and 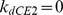. Set the AUTO axes to run from 0 to 0.3 along the x-axis and from −0.01 to 1.8 along the y-axis; (c) Bifurcation diagram of [E2F] with 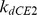 as a control parameter at 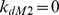 and 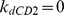. Set the AUTO axes to run from 0 to 5 along the x-axis and from −0.01 to 1.2 along the y-axis.

In order to further compare the repressions of miR449 on E2F by targeting three different transcripts named Myc, CycD-Cdk4/6 and CycE-Cdk2, the dynamical behaviors of E2F versus the inhibition rate of miR449 on these three transcripts are investigated numerically in Figure 10. Particularly, during studying the negative regulatory effect of miR449 on E2F by targeting Myc, we assume that 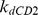 and 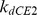, the inhibition rates of miR449 on CycD-Cdk4/6 and CycE-Cdk2, are zero. Then a bifurcation diagram in Figure 10(a) shows the function of E2F concentration corresponding to 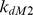, the inhibition rate of miR449 on Myc. Using the same idea, other two bifurcation diagrams respectively describing the dynamical behaviors of E2F level as a function on 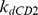 and 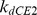 are shown in Figure 10(b) and (c).

Interestingly, these three bifurcation diagrams are quite distinct. Firstly, Figure 10(a) shows that E2F always settles on a high stable steady state without the regulation of miR449 (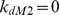). When the inhibitory effect of miR449 on Myc is introduced (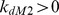), the high stable steady state of E2F decays rapidly with increasing 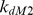. If the value of 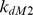 is limited between two supercritical Hopf bifurcation values 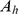 and 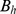, the high stable steady state of E2F losses its stability and sustained oscillations of E2F appears. When 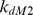 keeps on going out of the range, these oscillations vanish and then E2F steps into its low stable steady states. In Figure 10(c), under the regulation of miR449 on E2F by targeting CycE-Cdk2, the concentration of E2F always stays at a stable state, which descends quickly and then tends to a horizontal level. However, in Figure 10(b), the high stable level of E2F decreases gradually at the beginning of introducing repression of miR449 on E2F by targeting CycD-Cdk4/6. As the 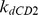 increases, E2F steps into a bistable range between two subcritical Hopf bifurcation values 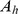 and 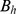. Different from the case of monostability in Figure 10(a) and (c), where a lower inhibition rate corresponds to a higher E2F concentration and a higher inhibition rate corresponds to a lower protein concentration due to the negative regulation mediated by miR449, when the inhibition rate lies between the two subcritical bifurcation points 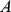 and 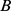, despite the negative regulation mediated by the miR449, a higher inhibition rate may correspond to a higher E2F concentration or a lower inhibition rate may also correspond to a lower E2F concentration, depending on the initial conditions. Thus, the numerical results and analysis suggest that the dynamical behaviors of the negative effects of miR449 on E2F by targeting three different transcript largely differ in the onset of oscillations, bistability and monostability, which needs to be verified experimentally. It is expected that the difference will generate a detailed and precise insight of miR449 mediated Rb-E2F pathway.

## Discussion

MiRNAs have been shown to function as integral components of a wide range of cellular processes including cellular growth, differentiation, and disease [11]–[15]. As such, miRNA dysregulation can have a profound effect on cancer development [21]–[24]. Previous studies have shown that miR449 is down-regulated or lost in gastric cells, testicular cancer cells as well as in lung adenocarcinoma cell line and possesses potential tumor suppressor function [21]–[24]. In this paper, to investigate the dynamical potential of miR449 in regulation of cell cycle, a mathematical model for Rb-E2F pathway mediated by miR449 are constructed based in part on the model proposed in Yao-Lee et al. (2008) and numerical simulation and dynamical analysis are performed. By comparing the dynamical behaviors of E2F pathway deregulation and regulation of miR449, we theoretically verified that the miR449 plays a critical role in regulating the cell cycle progression and provides a twofold safety mechanism to avoid excessive E2F-induced unrestricted proliferation by cell cycle arrest and apoptosis. Moreover, numerical simulation suggests that the mechanisms of the negative regulation of miR449 on three different transcripts are quite distinctive which needs to be verified experimentally.

The results in this paper turned out that the model without miR449 has several disadvantages than the one with miR449:

- Model without miR449 is risky. As 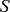 increases, E2F concentration quickly transit to a high level that will induce unrestricted proliferation. However, the model with miR449 is safer. It is the effective inhibition of E2F by miR449 that can prevent this abnormal proliferation by inducing cell cycle arrest or irreversible apoptosis.
- Model without miR449 has little practical significance. The periodic orbit corresponding to the normal cell proliferation is unstable in Figure 2 and 3, that is, the orbits can scarcely be reached in real environment. But the model with miR449 is proper in Figure 4 and 5 because the limit cycle corresponds to the cell proliferation are stable.
- Model without miR449 is less robust. It can be seen that the oscillatory range of the model without miR449 is smaller, indicating that the model under regulation of miR449 is more robust. Thus, miR449 has the ability to enhance the fidelity, robustness and flexibility in temporal regulation.

The study of Rb-E2F pathway mediated by miR449 may help us to analyze the whole cell cycle process mediated by other miRNAs more easily and may even provide an new insight for therapeutic manipulation in the treatment of cancer. A better knowledge of the miRNAs mediated gene regulation is also of interest for the bio-engineering or artificial control of specified components, interactions, and even network functions [1], [2], [21]–[24].

Certainly, besides Rb-E2F pathway mediated by miR449, more complex models will be needed soon to accommodate the complexity of miRNAs in gene regulatory networks. For example, further improvements can be done to the present model in future. Firstly, for simplicity, we only considered the cell cycle of 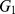 to 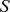 phase in this study. Further exploration on the whole cell cycle process mediated by miRNAs still needed be researched intensively, which will provide a more comprehensive view on how cell cycle are regulated by miRNAs. Secondly, how do miR449 and miR34 cooperatively fine tune the E2F and p53 pathways in the case of DNA damage is another interesting and challenging problem [21]. After DNA is damaged, p53 and E2F coordinate cell cycle progression and cell fate decision including growth arrest, DNA repair and apoptosis [47]. Moreover, p53 induces miR34 and E2F increases the levels of miR449 [21], [22], [26]. The most exciting aspect is miR449 and miR34 each repress E2F, but promote p53 activity, allowing efficient cross-talk between two major DNA damage-responsive gene regulators, which may thus represent a determinant for therapeutic manipulation in the treatment of cancer by irradiation or chemotherapy [21]. Therefore, to obtain a comprehensive and coherent picture of the regulatory roles of miRNAs are urgent, and such important aspects still need to be considered.

## Materials and Methods

Numerical simulation and bifurcation analysis of the ODEs in our paper were carried out with XPPAUT, a software program freely downloadable from http://www.math.pitt.edu/ 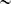
bard/xpp/xpp.html. XPPAUT can automatic detect bifurcations of fixed points and limit cycles. It is base upon the following strategy:

- Use Sing pts Go command to compute stable steady point for the system. XPPAUT will used this point as initial conditions for drawing bifurcation diagrams.
- Use the Axes command to tell XPPAUT what parameters are varied and what is to be plotted, as well as the range of the graphs.
- Use the Numerics command to define all the AUTO numerical parameters such as the direction, step size, and so on.
- Use the Run command to run the bifurcation. Grab the Hopf bifurcation and track the periodic orbits.

Values for the rate constants in our model are shown in Table 1 unless specified elsewhere. They were chosen based on the literature whenever possible [25], and on the biochemical constraints [21], [22], [26], [42] to give simulations and bifurcation diagrams that are consistent with known behaviors of miR449 and Rb-E2F pathway. XPPAUT codes including all the parameter sets are available in the Supporting Information Section.

## Supporting Information

File S1
**In order to calculate the bifurcation diagram of [E2F] and [CycE-Cdk2] with S as a control parameter at** 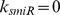 **, we provide XPPAUT code of** **Figure 2** **and** **3** **in this Supporting Information File S1.**
(ODE)

File S2
**To obtain the bifurcation diagram of [E2F] and [CycE-Cdk2] with S as a control parameter at** 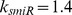 **, XPPAUT code of** **Figure 4** **and** **5** **is available in this Supporting Information File S2.**
(ODE)

File S3
**To calculate time courses of [E2F] and [CycE-Cdk2] at** 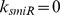 **and** 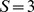 **, we provide XPPAUT code of** **Figure 6(a)** **in this Supporting Information File S3.**
(ODE)

File S4
**In order to obtain time courses of [E2F] and [CycE-Cdk2] at** 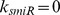 **and** 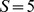 **, XPPAUT code of** **Figure 6(b)** **is provided here.**
(ODE)

File S5
**To calculate time courses of [E2F], [CycE-Cdk2] and [MiR449] at** 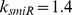 **and** 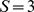 **, we provide XPPAUT code of** **Figure 7(a)** **in this Supporting Information File S5.**
(ODE)

File S6
**To obtain time courses of [E2F], [CycE-Cdk2] and [MiR449] at** 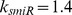 **and** 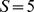 **, XPPAUT code of** **Figure 7(b)** **is available in this Supporting Information File S6.**
(ODE)

File S7
**In order to plot the bifurcation diagram of [E2F] with** 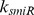 **as a control parameter at** 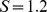 **, we provide XPPAUT code of** **Figure 8(a)** **in this Supporting Information File S7.**
(ODE)

File S8
**In order to obtain the bifurcation diagram of [E2F] with** 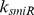 **as a control parameter at** 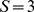 **, in this Supporting Information File S8 we provide XPPAUT code of** **Figure 8(b)** **.**
(ODE)

File S9
**In order to calculate the bifurcation diagram of [E2F] with** 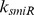 **as a control parameter at** 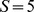 **, XPPAUT code of** **Figure 8(c)** **is provided in this Supporting Information File S9.**
(ODE)

File S10
**To obtain time courses of [E2F], [CycE-Cdk2] and [MiR449] at** 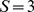 **and** 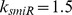 **, XPPAUT code of** **Figure 9(a)** **is include in this Supporting Information File S10.**
(ODE)

File S11
**To calculate time courses of [E2F], [CycE-Cdk2] and [MiR449] at** 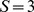 **and** 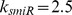 **, we provide XPPAUT code of** **Figure 9(b)** **in this Supporting Information File S11.**
(ODE)

File S12
**In order to obtain the bifurcation diagram of [E2F] with** 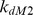 **as a control parameter at** 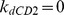 **and** 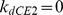 **, we provide XPPAUT code of** **Figure 10(a)** **here.**
(ODE)

File S13
**To plot the bifurcation diagram of [E2F] with** 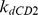 **as a control parameter at** 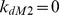 **and** 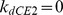 **, XPPAUT code of** **Figure 10(b)** **is provided in this Supporting Information File S13.**
(ODE)

File S14
**To calculate the bifurcation diagram of [E2F] with** 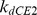 **as a control parameter at** 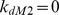 **and** 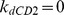 **, in this Supporting Information File S14 we include XPPAUT code of** **Figure 10(c)** **.**
(ODE)
